# *Behavioral and Brain Functions* at 15

**DOI:** 10.1186/s12993-020-00170-w

**Published:** 2020-10-23

**Authors:** Wim E. Crusio

**Affiliations:** University of Bordeaux and CNRS, Pessac, France

Fifteen years ago, on April 22, 2005, the first articles published by the new journal *Behavioral and Brain Functions* (*BBF*) were made available online [1]. The journal was the fruit of the enthusiastic efforts of Terje Sagvolden, who would be editor-in-chief until his untimely death in 2011 [2]. To the best of my knowledge, *BBF* was the first behavioral neuroscience journal that was online-only and open-access (OA). While this may seem to be quite out-of-the-ordinary nowadays, 15 years ago this was an important innovation. Many journals were still paper-only and just a short time before it had become clear that non-paper, “electronic” journals were eligible to be included in MEDLINE/PubMed or the Science Citation Index, or could obtain an impact factor. And although more general online-only OA journals had been established a few years earlier, it was not at all clear whether enough authors would be willing to forego paper and submit to the fledgling journal.

However, Terje’s instinct that the time was ripe for an OA journal in the field of behavioral neuroscience proved to be correct and, from the start, the journal received many diverse first rate submissions. By now, 15 years later, the journal has established itself as a well-respected voice in the field, publishing a variety of high-quality articles that are regularly cited in other journals, as shown by its solid current impact factor of 2.125.

From the beginning, the journal published research on a diversity of organisms and this has continued to the present day. Indeed, recent published articles have covered research on fruit flies [3, 4], zebrafish [5], the obligatory rodents [e.g., 6, 7], and humans [8, 9]. Also, many different techniques are used in the articles that we publish, a consequence of the interdisciplinary and multidisciplinary nature of our field, which draws from psychology, psychiatry, ethology, neuroscience, genetics, developmental biology, and more. As the journal’s aims state, we publish research providing insight into the neurobiological mechanisms underlying behavior and brain function, or dysfunction, excluding only studies that are purely clinical.

Over the years, the field has experienced significant growth, as the notion that the most important aspect of brain science is how the brain’s output gets translated into behavior gained more and more traction. To paraphrase Theodosius Dobzhansky: Nothing in neuroscience makes sense except in the light of behavior. As a result, more and more neuroscience studies nowadays include at least some behavior.

While *Behavioral and Brain Functions* covers the whole field of behavioral neuroscience, we are especially interested in covering work that addresses some of the key issues that the field is facing. Two of the most pressing issues are the validation of behavioral concepts [10] and the (related) replicability crisis [11]. Some of the failures to replicate behavioral results may be due to laboratory-specific conditions. For example, Crabbe and colleagues showed that even rigorously standardized tests sometimes give widely varying results in different laboratories [12]. (It should be stressed here that they also showed that *most* tests were eminently replicable).

Other failures to replicate, however, lay on a whole different level. From the results of behavioral tests, both in animals and in humans, we infer behavioral concepts such as spatial learning, anxiety, behavioral despair, etc. However, often enough tests that purportedly tap into the same behavioral construct do not correlate with each other [e.g., 13], indicating that perhaps our inferences are missing something. More research on what exactly our behavioral tests are measuring is urgently needed and Behavioral and Brain Functions hopes to play a role in disseminating such work, be it in the form of research articles or reviews.

Finally, at the occasion of our 15th anniversary, I would like to express my sincere gratitude to all those who have worked tirelessly to get the journal where it currently is: my predecessors as editors-in-chief, those who have served as associate editors, and the members of the editorial board. Many thanks are also due to our authors for submitting their work to us for consideration for publication, but above all I want to recognize our unheralded reviewers, who selflessly donate their time, effort, and expertise to maintain the quality of our journal.

While celebrating the successful past 15 years, we look forward with anticipation to the future and further growth of our journal!
